# Medical and Physical Intelligence

**Published:** 1821-01

**Authors:** 


### Medical and Physical Intelligence.

TRANSACTIONS OF SCIENTIFIC SOCIETIES.

*ROYAL SOCIETY of London.—Nov. 9.* A PAPER, by Sir E. HOME, was read, entitled "*On the Black Rete Mucosum of the Negro, being a Defence against the Scorching Effects of the Sun's Rays.*" The author began by stating some observations which had induced him to form the opinion that the scorching effects of the sun's rays are produced, not merely in virtue of their heating power, but by the joint agency of their heat and light. To verify this opinion, he made several experiments which showed that the face and hands may be exposed to a temperature of 100°, or even 120°, without pain being produced, provided the light be excluded: but that if the same, or even an inferior degree, of heat be produced by the direct rays of the sun, the parts are scorched, and blisters are produced. This effect he found to be completely prevented by covering the hand or face with black kerseymere; and the same purpose is attained by the black rete mucosum of the negro. In those cases when a black covering was superimposed, perspiration came on; and the same takes place on the skin of the negro, when exposed to the direct rays of the sun.

The author observed, also, that the eyes of those animals exposed to the strong light of the sun are furnished with a black pigment, apparently for the same purpose; while others, which are abroad by night, and consequently not needing such a protection, are not provided with it.

*Nov.* 16.—A paper, by Sir H. DAVY, was read, entitled "*On the Magnetic Effects produced by Electricity.*"

On repeating Oersted's experiments, the author stated that, with a voltaic battery of 100 four-inch plates, the south pole of a magnetic needle placed under the communicating wire of platinum, the positive end of the apparatus being on the right, was strongly attracted by the wire, which was shown to be itself magnetic by its power of attracting steel-filings. The wire also was found to communicate permanent magnetism to steel bars attached to it transversely; while such bars placed parallel to the wire, were only magnetic during their connection with the apparatus.

Actual contact, however, of the steel wires with the platinum, or other metal forming the conductor, was found not to be necessary; for magnetism was imparted to a needle placed transversely to the connecting metal, but at some distance from it.

Sir H. next related some experiments, showing that the magnetic power is proportionate to the quantity of electricity passing through a given space, without any relation to the transmitting vessel; and that, the finer the wires, the stronger the magnetic effect.

The author found that an analogous effect was produced by the discharge of a Leyden phial through a wire; and, by passing the discharge of a Leyden battery of seventeen square feet through a silver wire with a steel bar transversely attached to it, of two inches in length, the latter became powerfully and permanently magnetic. The same effect was produced at a distance of five inches through air, water, and even through thick plates of glass.

When several wires parallel to each other formed part of the same circuit, each became similarly magnetic to the single wire, and the opposite ends of each wire were in different magnetic states, and consequently attracted each other. When two voltaic batteries were placed parallel to each other, the positive end of the one being opposite to the negative end of the other, and transmitting their electricity through two wires, such wires repelled each other, because the opposite ends were in similar electrical states.

The Copley medal has been voted to Professor OERSTED for his important discovery of the connection between electricity and magnetism.

---

WE extract the following aphorisms respecting the treatment of *Puerperal Fever*, from the Inaugural Thesis of a Dr. LEGOUAIS, maintained during the last year before the Faculty of Paris; not because they are *new*, for they are merely principles which Dr. ARMSTRONG had previously established in the most satisfactory manner, but because the author was a pupil of Professor CHAUSSIER, and for a long time a resident student at the Lying-in Hospital *La Maternité*. They are, therefore, deduced from very extensive observation, and are, no doubt, the precepts of the excellent Professor whom we have just named. Such coincidences of opinion, especially when the extraordinary acquirements, remarkable acumen of observation, and very rare intellectual talents, of Chaussier are considered, cannot fail to give a great degree of interest to the following paragraphs.

"1. Excepting in the cases where there exist some particular and manifest contra-indications to the use of local, and especially of general, evacuation of blood, in the treatment of puerperal peritonitis, this measure should be the first to be employed.

"2. In order that bleeding may be followed by success as certainly as we can ever expect in the treatment of diseases, it should be employed in the first stage,—that is to say, in by far the greater majority of cases, before the termination of the first day of the disease; and, moreover, that a sufficiently abundant quantity of blood should be taken away to destroy at once all the violence of the malady.

"3. When employed not in conformity with those two essential conditions, sanguineous evacuations, far from being useful, always become very injurious to the patient, by destroying her strength and the resources of nature, without having a durable influence against the disease.

"4. In the cases,—and these are the most common instances,—where constipation is one of the attendant circumstances of the disease, another indication, not less pressing for its fulfilment, is joined with that of blood-letting, which is to excite alvine evacuations, by gentle, or even by somewhat active, purgatives, and thus to maintain a sort of artificial diarrhœa during the whole of the duration of the disease; a duration which, in the greater number of cases where the termination is favourable, does not exceed the space of from two to four days.

"5. When a diarrhœa exists from the commencement of the disease, nothing should be done to suppress it when it is moderate: when, on the contrary, it appears excessive, none but mild and soothing means should be employed, such as emollient clysters, mucilaginous drinks, and some opiates; and we should by no means add to the irritation by the administration of astringents."

---

We select the following theorems from the Inaugural Thesis of Dr. Ratier, sustained last year before the Faculty of Paris. They are, in almost every instance, only conformable with some of the inferences comprised in the excellent Treatise on the Blood of Mr. Thackrah; but it can never be considered uninteresting to witness a similarity of results from the researches of different persons on an important and practical subject relating to medicine. Alluding to the *buffy coat* on the blood, Dr. Ratier says—

"1. It is foreign to the state of perfect health, and equally so to bilious, mucous, typhoid, and asthenic, diatheses; to passive hemorrhages; and to nervous and organic affections.

"2. It appertains to the state of plethora, but only when it exists with a disposition to inflammations in general, and especially to those of the serous and parenchymatous membranes. It may exist without general inflammatory disposition in the system, when there is a very intense local inflammation.

"3. Its presence, and, when it exists, its thickness and density, depend on the intensity of the inflammation, on the largeness of the incision, on the form and rapidity of the jet, and, lastly, on the form of the vessel in which the blood is received.

"4. It is always composed of fibrine,—at least of a great proportion of that substance.

"5. It always manifests itself in a direct ratio with the intensity of the inflammation.

"6. Its presence, in connection with the other symptoms of inflammation, confirms the diagnostics of that affection.

"7. The absence of it should not be considered a reason for not resorting to blood-letting, if it be otherwise indicated.

"8. Its presence without clearly evident signs of inflammation should rouse the attention of the practitioner, and render him very circumspect if he employs tonics.

"9. Some anomalies may occur in respect to its existence, and in some cases, even with all the apparently requisite conditions, it is not manifest; but then we constantly find an extraordinary degree of density of the coagulum."

---

We transcribe the following table, by Dr. John Forbes, of Penzance, from a late Number of Dr. Thomson's *Annals of Philosophy*:—

### Comparative View of Mean Temperature at different Places in Great Britain.

| | Time of observation. | Mean Temperature. Winter. | | | Spring. | | | Summer. | | | Autumn. | | | Mean Temperature. | | | | | Var. of Temper.‡‡ | |
|---|---|---|---|---|---|---|---|---|---|---|---|---|---|---|---|---|---|---|---|---|
| | | Nov. | Dec. | Jan. | Feb. | March | April | May | June | July | August | Sept. | O | Winter. | Spring. | Summer. | Autumn. | Whole year. | Annual range. | Mean variation of successive months. |
| | a. m. | | | | | | | | | | | | | | | | | | | |
| Isle of Wight, 10 yrs. (1809—1818)* | 9 | 44 | 39 | 37 | 41 | 44 | 46 | 56 | 62 | 65 | 62 | 58 | 51 | 40 | 43 | 61 | 57 | 50 | 25 | 4.66 |
| † Penzance, 11 yrs. (1807—1817) ‡ | 8† | 45 | 42 | 40 | 42 | 43 | 46 | 53 | 57 | 59 | 59 | 56 | 50 | 42 | 43 | 56 | 55 | 49 | 19 | 3·16 |
| Sidmouth, 2 yrs. (1813—1814) § | 8 | 44 | 41 | 34 | 40 | 43 | 50 | 54 | 60 | 64 | 63 | 59 | 50 | 39 | 44 | 59 | 57 | 50 | 30 | 5·00 |
| Exeter, 5 yrs. (1814—1818) ‖ | 8 | 43 | 37 | 34 | 38 | 41 | 44 | 51 | 57 | 59 | 57 | 53 | 47 | 38 | 41 | 55 | 52 | 46 | 25 | 4·16 |
| Gosport, 4 yrs. (1816—1819) ¶ | 8 | 43 | 37 | 39 | 39 | 41 | 46 | 52 | 60 | 62 | 61 | 57 | 49 | 39 | 42 | 58 | 55 | 48 | 25 | 4·16 |
| London, 3 yrs. (1817—1819) ** | 8 | 45 | 36 | 38 | 39 | 40 | 45 | 52 | 59 | 61 | 60 | 56 | 48 | 39 | 41 | 57 | 54 | 48 | 25 | 4·16 |
| Kinfauns, 6 yrs. (1813—1818) †† | 8 | 39 | 34 | 32 | 36 | 37 | 42 | 48 | 54 | 57 | 55 | 51 | 44 | 35 | 38 | 53 | 50 | 43 | 25 | 4·16 |

* Original Journal, by — Kirkpatrick, Esq.

† I have stated the time here at eight, *a. m.* but the observations were taken at seven, *a. m.* in all the months except December and January. Of course, some allowance ought to be made on account of the earlier (and colder) period of observation at Penzance.

‡ Original journal, by Thomas Giddy, Esq.

§ Dr. Clarke, in *Annals of Philosophy.*

‖ Original journal, by E. P. Pilcher, Esq.

¶ Dr. Burney, *in Annals of Philosophy.*

** Mr. Cary, in *Philosophical Magazine.*

†† Thomson's *Annals.*

‡‡ These two columns are formed from the mean temperature as given in the preceding part of the table.

### REPORT OF DISEASES.

THE diseases most prevalent during the last month have differed but little from those we mentioned in our Report for the one immediately preceding; a circumstance easily accounted for by the similar states of the atmosphere during these periods. Rheumatism continues to abound in all its forms, and we have recently seen three cases in which the disease appeared to be translated to the chest: in two of these instances, the attack of pain in the region of the heart, accompanied with much anxiety and disturbed respiration, followed so closely the subsiding of the pain and swelling of the limbs, as to leave no doubt of its real nature. We have nothing to add to what we have already stated regarding the treatment of rheumatism, except that we have tried the *vinum seminum colchici* in eight or ten cases, without being able to perceive any advantage possessed by this form over the one in more general use. Of this, however, we are quite satisfied, that it acts more readily upon the bowels,—rather severe purging having in several instances followed its exhibition even in moderate doses. When this drug does irritate the bowels, the evil is best remedied by magnesia, or a few grains of ipecacuanha, two or three times a-day. We are likewise convinced that it is a mistake to suppose that this operation upon the bowels is in any way connected with its proper effects upon the constitution; and, consequently, that it is best to check it immediately, upon the same principle that we give opium when diarrhœa arises during the exhibition of the blue-pill. Both the forms of colchicum seem to us equally efficacious remedies in those chronic affections of the mucous membrane of the lungs in which the last month has been abundantly prolific.

Measles, which during the two preceding months had been epidemic, are now again disappearing, though some cases still occur. The sequelæ of the disease, however, are by no means overcome, the cough in many proving obstinate, and bronchitis ensuing in some cases.

Some severe cases of diarrhœa, attended with distressing head-ach and nausea, have likewise occurred: these have seemed to be caused by the abuse of malt-liquors.

Bronchitis, especially in children, has been more prevalent, we think, during the last two or three weeks than heretofore during this season; and we have witnessed a few cases of croup; two of which, affecting children of the same family, are very remarkable, in the disease passing into a chronic form, and being attended with paroxysms of such loud croaking that respiration may be heard in another room than that in which the children may happen to be. These paroxysms come on at irregular periods, without evident exciting causes, and continue for about half an hour; the breathing in the intervals can hardly be heard. This affection has been of two months' duration, in its present form, according to the mother's account. We did not see the patients until a few days since. The future history of the cases shall not be forgotten in our Reports.

Whooping-cough is very common. The disease has generally appeared as simple bronchitis in the first instance; and then, after from two or three to ten days, the whooping has come on. We have employed the colchicum in several cases of this affection, with results that make us consider that in this and the prussic acid we possess new and very powerful means of combating that disease.

---

### METEOROLOGICAL JOURNAL.

By Messrs. William Harris and Co. 50, Holborn, London.

*From November 20 to December* 19, *inclusive.*

| Day of Month. | Moon. | Rain gauge | THERM. | | | BAROM. | | DeLuc's Hygro. | | WIND. | | ATMOSPHERIC VARIATION. | | |
|---|---|---|---|---|---|---|---|---|---|---|---|---|---|---|
| Nov | | | | | | | | | | | | | | |
| 20 | ○ | | 45 | 48 | 36 | 30·05 | 30·09 | 61 | 60 | S | SW | Fog | | Cloud. |
| 21 | | ‘03 | 40 | 45 | 39 | 30·00 | 29·85 | 62 | 60 | S | WSW | Fog | Rain | Cloud. |
| 22 | | ‘20 | 42 | 45 | 39 | 29·63 | 29.47 | 62 | 64 | SW | SW | Fine | Rain | |
| 23 | | ‘19 | 43 | 47 | 35 | 29·50 | 29·59 | 63 | 62 | S | WSW | Rain | | Fine |
| 24 | | ‘05 | 39 | 41 | 37 | 29·63 | 29·67 | 62 | 60 | SE | ESE | Fog | | Cloud |
| 25 | | ‘06 | 41 | 43 | 35 | 29·65 | 29·62 | 62 | 62 | SSE | S | Cloudy | | |
| 26 | | ‘03 | 40 | 43 | 38 | 29.77 | 29 95 | 61 | 62 | SE | E | Fine | | Cloud. |
| 27 | ☽ | | 42 | 45 | 40 | 29·94 | 30·02 | 61 | 61 | E | E | Cloudy | Fine | |
| 28 | | | 43 | 47 | 40 | 30·15 | 30·22 | 60 | 60 | E | ENE | Cloudy | Fine | |
| 29 | | | 42 | 45 | 37 | 30·26 | 30·30 | 61 | 59 | E | NE | Cloudy | | |
| 30 | | ‘02 | 41 | 45 | 35 | 30·26 | 30·20 | 60 | 60 | N | NW | Fine | | |
| Dec | | | | | | | | | | | | | | |
| 1 | | | 39 | 45 | 35 | 30·16 | 30·07 | 60 | 60 | NW | W | Fog | Cloud. | |
| 2 | | | 37 | 45 | 36 | 30·05 | 30·10 | 60 | 60 | W | WNW | Fog | Fine | |
| 3 | | | 39 | 47 | 38 | 30·10 | 30·00 | 62 | 63 | WSW | WNW | Rain | Fine | Cloud. |
| 4 | | | 40 | 47 | 39 | 30·00 | 30·01 | 65 | 65 | W | WSW | Cloudy | | |
| 5 | ● | | 43 | 49 | 39 | 29·97 | 29·91 | 63 | 61 | W | WNW | Fine | | |
| 6 | | ‘15 | 45 | 50 | 40 | 30·10 | 30·09 | 60 | 64 | SE | E | Fog | Clou d. | |
| 7 | | | 48 | 56 | 40 | 30·05 | 30·11 | 63 | 63 | W | W | Fog | | |
| 8 | | | 48 | 52 | 45 | 30·16 | 30·21 | 61 | 62 | W | W | Fine | | |
| 9 | | | 50 | 52 | 50 | 30 20 | 30·12 | 59 | 60 | WSW | WSW | Cloudy | Fine | |
| 10 | | ‘13 | 52 | 53 | 50 | 30·04 | 29·98 | 61 | 61 | SW | WSW | Rain | | Cloud. |
| 11 | | | 52 | 53 | 50 | 29·88 | 29·88 | 61 | 61 | WSW | SW | Fine | | Cloud. |
| 12 | ☾ | ‘20 | 52 | 55 | 46 | 29·63 | 29·55 | 65 | 62 | SW | SW | Rain | | |
| 13 | | ‘52 | 50 | 44 | 30 | 29 56 | 29·73 | 62 | 60 | NNE | NE | Rain | | Fine |
| 14 | | ‘13 | 32 | 35 | 30 | 29·88 | 29·95 | 56 | 57 | NNE | ENE | Fine | | Sleet |
| 15 | | | 30 | 33 | 30 | 30·00 | 29·88 | 57 | 55 | SE | SE | Cloudy | | Fine |
| 16 | | ‘09 | 32 | 35 | 33 | 29·73 | 29·60 | 57 | 60 | SE | SE | Cloudy | | Rain |
| 17 | | ‘14 | 35 | 38 | 40 | 29·68 | 29·93 | 61 | 63 | E | E | Sn. & Ra. | Rain | Cloud. |
| 18 | | ‘05 | 43 | 48 | 41 | 30·10 | 30·20 | 65 | 65 | SE | S | Cloudy | Rain | Fine |
| 19 | ○ | ‘09 | 46 | 49 | 42 | 30·26 | 30·34 | 65 | 65 | SSW | W | Rain | | Cloud. |

The quantity of rain fallen in the month of November, is 1 inch and 40-100ths.

